# Infection Dynamics and Clinical Signs of Bluetongue Virus Serotype 3 in Cattle and Transmission to *Culicoides* Vectors

**DOI:** 10.1155/tbed/9342380

**Published:** 2026-10-31

**Authors:** Kerry Newbrook, Laura Jones, Emmanuel Obishakin, Martin Ashby, Simon Gubbins, Carrie Batten, Christopher Sanders

**Affiliations:** ^1^ Non-Vesicular Reference Laboratory The Pirbright Institute Woking GU24 0NF UK pirbright.ac.uk; ^2^ Entomology Group The Pirbright Institute Woking GU24 0NF UK pirbright.ac.uk; ^3^ Transmission Biology Group The Pirbright Institute Woking GU24 0NF UK pirbright.ac.uk

**Keywords:** biting midge, bluetongue disease, bovine, BTV, duration of infection, isolation, viremia

## Abstract

Outbreaks of bluetongue (BT) virus (BTV) serotype 3 (BTV‐3) have been reported across Europe since late 2023, causing significant levels of disease in sheep, goats and cattle. The infection dynamics and clinical disease severity in the UK cattle and the infection rate of European *Culicoides* vectors to this BTV‐3 strain have yet to be investigated. Four Holstein‐Friesian calves were infected with BTV‐3 UKG2023/04 using a host:virus:vector transmission model with *Culicoides sonorensis* biting midges. Clinical signs, gross pathology, infection kinetics, duration of infectious viraemia in the blood and host immune response were assessed. The potential for onwards BTV transmission from infected calves to colony‐reared and the UK field‐caught *Culicoides* biting midge vectors was quantified over the study period. All four calves were infected with BTV‐3, even when few infected *Culicoides* had acquired a blood meal. Infected calves experienced mild clinical signs and transient elevation in rectal temperatures, presenting lower disease severity than previously reported in European cattle in the field. BTV infection, measured through the detection of viral RNA by real‐time RT‐PCR of the blood, peaked at 6–10 days post‐infection (dpi) for all individuals. Infectious BTV‐3 was isolated from the blood of three infected calves up to 56 dpi, although the consistency with which BTV was isolated declined after 24 dpi. Protective neutralising antibodies against BTV VP2 were first detected in calves from between 10 and 16 dpi. BTV RNA was detected in colony‐reared and the UK field‐caught *Culicoides* that had fed on infected calves at peak viraemia (6–8 dpi) and later in infection (42 and 55 dpi). Estimates of the vector competence of the *Culicoides obsoletus* group for BTV‐3 were low (0.68%). Our data indicate that whilst cattle are less likely than sheep to exhibit clinical disease following infection with BTV‐3, the duration of infectious viraemia observed may permit transmission to *Culicoides* vectors for several weeks post‐infection.

## 1. Introduction

Bluetongue (BT) is an infectious, non‐contagious, haemorrhagic arboviral disease of domestic and wild ruminants caused by BT virus (BTV) (family: Sedoreoviridae, genus: *Orbivirus*) which is predominantly transmitted by *Culicoides* biting midges (Diptera: Ceratopogonidae) [1]. Infection with BTV can be characterised by facial oedema, fever, conjunctivitis, reddening of the mucosal membranes, nasal discharge, depression and lameness [2]. Disease severity varies with virus serotype/strain, host species and breed. The greatest disease burden has been reported in sheep, specifically fine‐wool breeds, which can exhibit severe clinical signs accompanied by high mortality rates [3]. Cattle can become infected with BTV and typically exhibit few, if any, clinical signs of disease yet play an important role in transmission [3]. Over the last few decades, the geographical distribution of BTV has shifted, with new serotypes and strains emerging and spreading across Europe [4]. Northern Europe has experienced outbreaks of multiple BTV serotypes since 2006, with the outbreak of BTV‐8 between 2006 and 2010 causing the most significant economic losses [5, 6]. The most recent example has been the emergence and spread of BTV serotype 3 (BTV‐3) since 2023 [7].

BTV‐3 emerged in the Netherlands in September 2023 and spread rapidly, affecting over 5000 sheep flocks and cattle herds throughout the country, with subsequent cases identified across Europe including in Belgium, Germany and the UK [8]. In the Netherlands, severe clinical signs were described in the field in sheep infected with BTV‐3. In BTV‐3‐infected cattle, signs were less severe than in sheep, although a decreased milk yield and a national noticeable increase in mortality were reported [8]. BTV‐3 was initially identified in the UK in November 2023 through annual active surveillance on the south coast of England. This marked the first incursion and subsequent autochthonous transmission of BTV in the UK for more than 15 years, resulting in livestock movement restrictions on and around affected farms, impacting welfare plus national and international livestock trade.

In contrast to the outbreak in the Netherlands, no clinical signs of BT disease were reported in any of the UK BTV‐3 cases detected in 2023, although a relatively small number of animals (119 cattle and 7 sheep) were found to have been infected through active surveillance [9]. In 2024, BTV‐3 re‐emerged in northern Europe, extending its range to affect at least 17 countries, including the UK, where cases were reported across south and east England [10]. In the UK during the 2024–2025 vector season, 212 BTV‐3 positive cases were detected by RT‐qPCR in ruminants. BTV‐3 has since overwintered in the UK, re‐emerging in July 2025, with a further 232 cases detected in England and Wales [11]. BTV‐3 positive cases in the UK have largely been detected through active surveillance and testing of animals for movement, with few detections following reports of suspected clinical BT disease, with foci of disease in the southwest of England in 2025 [12]. Reproductive complications during lambing and calving, including calves born with neurological disease associated with BTV‐3 infection, have been reported [13]. In July 2026, the first BTV‐3 case was reported in the southwest and has been followed by a rapidly increasing number of reports of severe disease in BTV‐3 positive sheep and cattle [14].

In a previous in vivo study, BTV‐3‐infected UK‐native North Country mule sheep exhibited a range of clinical signs of BT disease, ranging from mild to moderate severity [15]. The sheep developed a persistent viraemia, where infectious BTV‐3 remained up to 24 and 28 days post‐infection (dpi) in the blood of two surviving individuals [15]. In cattle, longer viraemia periods have previously been described following infection with other BTV serotypes, with the infectious virus isolated several weeks post‐infection [16, 17]. A prolonged infectious viraemia combined with typically milder disease or asymptomatic infection may permit cattle to act as a sub‐clinical reservoir for transmission to *Culicoides* vectors. Importantly, the duration of the period of infectivity and transmission from cattle to *Culicoides* may act as a mechanism of overwintering of BTV between vector seasons within temperate regions [18]. The length of infectious viraemia following BTV‐3 infection in cattle, clinical severity and the susceptibility of native UK *Culicoides* species to BTV‐3 infection have yet to be determined.

Here, we infected four British Holstein‐Friesian calves with the UK BTV‐3 (UKG2023/04) strain via *Culicoides sonorensis* (Worth and Jones) midges to characterise the resulting clinical signs and disease severity, gross pathology, infection kinetics, host antibody responses and the duration of infectious viraemia in systemic blood. The duration over which BTV‐3‐infected calves remained infectious to naïve colony‐reared *C. sonorensis* and the UK field‐caught *Culicoides* species was assessed weekly from peak viraemia to the end of the study at 56 dpi.

## 2. Methods

### 2.1. Virus

BTV‐3 UKG2023/04 was previously isolated from the blood of a UK cow infected in 2023 and serially passaged four times through KC cells [KC4] to generate a virus stock (supernatant) for use in a previous in vivo study in sheep [15] and the current study.

### 2.2. Insect Vectors

Newly emerged adult *C. sonorensis* midges (PIRB‐S‐3 strain) were obtained from the colony at The Pirbright Institute [19, 20]. *Culicoides* were housed in 64 mm cardboard pots (Watkins and Doncaster, UK), sealed with fine netting and maintained at 25°C (70%–90% rH) prior to use in the study. Each mixed‐sex pot contained ~250 female *C. sonorensis*.

Field collection of adult *Culicoides* was conducted at two sites close to The Pirbright Institute (Woking, UK) throughout the study period. Down‐draught miniature blacklight (UV) Centers for Disease Control (CDC) light traps (model 912, John W Hock Co., Florida, USA) were used to collect adult host‐seeking *Culicoides*. Traps were placed at equine holdings and suspended at a height of ~1.5 m above ground. Each week, traps were set up at least 2 h before sunset and run overnight before the collection of live insects within 2 h of sunrise the following morning. Adult *Culicoides* were maintained in an environmentally controlled chamber (Aralabs, UK) at 25°C and 80% rH for 1–2 days prior to feeding on the calves. During maintenance, a 10% (w/v) sucrose solution was provided on cotton wool ad libitum and removed 24 h prior to feeding on the infected calves. Adult *Culicoides* from different collection sites were combined and transferred to 64 mm cardboard pots (Watkins and Doncaster, UK) sealed with fine netting ~2 h before feeding, with each pot containing 200– 400 females.

### 2.3. Cell Lines

KC cells, derived from 2‐day‐old *C. sonorensis* embryos [21], were maintained as previously described [22]. Vero cells were maintained at 37°C under humidified conditions (5% CO_2_), as previously described [23, 24].

### 2.4. In Vivo Study

Four female Holstein‐Friesian calves (6 months old) were maintained in the Pirbright Institute’s high‐containment animal facility. Prior to the study, all calves tested negative for BTV RNA by real‐time RT‐PCR and for antibodies in serum against the group‐specific BTV viral protein 7 (VP7) by ID Screen BT competitive ELISA (Innovative Diagnostics, France). Calves were group‐housed, free roaming and fed ad libitum hay and water, supplemented with grain pellets. Calves were acclimatised to the containment facility for 7 days prior to infection and monitored twice daily throughout the study.

BTV‐3 infection was attempted in the four calves with a natural in vivo host:virus:vector transmission model using *C. sonorensis* midges [15, 25, 26]. Low vector competence of *C. sonorensis* for this BTV‐3 strain [15] precluded infection of sufficient insects through oral feeding. Therefore, female *C. sonorensis* was intrathoracically (IT) inoculated with 0.2 µL BTV‐3 UKG2023/04 suspension (10^7^ TCID_50_ per millilitre), using a Nanoject II (Drummond, UK) microinjector and pulled glass needles as previously described [26]. Eighty IT‐inoculated midges were placed in each 64 mm netted cardboard pot (Watkins and Doncaster, UK) and incubated for 5–6 days at 25°C, 75%–85% rH with 10% (w/v) sucrose solution provided *ad libitum*. Sucrose was removed from the pots 24 h prior to feeding on the calves. To establish BTV‐3 infection, two pots of IT midges were held onto the shaved skin on the rump of each calf for 30 min to allow the surviving midges to feed. Following the feeding attempt, blood‐engorged *Culicoides* females were selected from each pot, under light CO_2_ anaesthesia and placed in collection tubes (Qiagen, UK). Blood‐fed females were selected as either fully engorged, replete individuals or ‘partially fed’, i.e., those with evidence of blood feeding or some engorgement. Collection tubes contained 200 µL RPMI 1640 media (Gibco, Thermo Fisher Scientific, UK) supplemented with 2% Penicillin‐Streptomycin (10,000 U/mL) (Gibco, UK) and amphotericin B (250 µg/mL) (Gibco, UK) and a 3 mm stainless steel bead (DeJay Distribution Ltd., UK) [27]. These were homogenised using a tissue lyser (Qiagen, UK) at 25 Hz twice for 30 s each before increasing the media volume to 1 mL and subsequently processed to detect viral RNA as described below. The number of BTV‐infected *C. sonorensis* that blood fed on each calf was determined as previously described [24, 26].

Clinical assessments for signs of BT‐associated disease were recorded daily from 2 to 56 dpi, and clinical scores were determined for each calf as previously described for sheep [15]. Rectal temperatures were recorded daily, with individuals’ baseline temperature calculated from an average of the −1 to 0 dpi readings. Raised temperatures and fever were defined as *a* ≥ 1°C and ≥ 1.5°C increase above individual baseline temperatures, respectively.

Whole EDTA and clotted blood were collected from the jugular vein of each calf prior to infection (−1 dpi) and then daily between 2 and 10 dpi (except for clotted blood at 3 dpi), after which blood was collected every other day until 56 dpi with additional collections at 21, 29, 35, 43, 49, and 55 dpi. Clotted blood was centrifuged at 2000 *g* for 10 min at 4°C, and serum was collected and stored at −80°C until use.

Calves were euthanised at the end of the study (56 dpi) through intravenous anaesthesia overdose with pentobarbital (140 mg/kg) and confirmed through permanent cessation of circulation. Full post‐mortem examinations were conducted on all calves, and any gross pathological changes were recorded. Tissue samples were collected from each calf at post‐mortem, homogenised (20% [w/v] in PBS) for 3 min and processed for the presence of BTV RNA as previously described [15, 28].

### 2.5. Infection Rate of *Culicoides* Midges

Naïve *C. sonorensis* (1–4 days post‐emergence) and UK field‐caught *Culicoides* spp. were blood‐fed on each calf at weekly intervals post‐infection to investigate the BTV infection rate as an indicator of the potential for onwards transmission, as previously described [24, 26]. Field‐caught *Culicoides* and colony *C. sonorensis* were fed on calves at 6 (field only), 7, 14, 21, 28, 35, 42, 49, and 55 dpi, with additional feedings of *C. sonorensis* only attempted at 8, 15, 22, 36, and 50 dpi. After feeding on infected calves, *Culicoides* were anaesthetised under light CO_2_ and fully blood‐engorged females were selected and incubated for 8 days as described above to complete the extrinsic incubation period. Following incubation, *Culicoides* were dispatched by placing at −80°C for at least 1 h before placement of individuals in collection tubes for homogenisation, RNA extraction and viral detection as described above. The number of surviving *Culicoides* following 8 days of incubation was recorded. *Culicoides* which had not survived incubation were identified through their desiccated appearance and were not selected for processing. A subset of ~8–16 fully engorged *C. sonorensis* fed on each calf at each feeding time point was collected and immediately frozen at −80°C prior to homogenisation, RNA extraction and real‐time RT‐PCR to quantify virus uptake by midges at the time of feeding (prior to incubation). Field‐caught *Culicoides* were morphologically identified to the species level by wing patterns under a dissecting microscope (Leica M26, Leica Microsystems, Germany) using published keys [29–31]. *Culicoides obsoletus* (Meigen) and *Culicoides scoticus* (Downs and Kettle) were grouped together as the *C. obsoletus* group. All field‐caught *Culicoides* that fed on infected calves at 7 and 14 dpi were dissected to assess the likelihood of dissemination of virus to the head, indicative of transmissible salivary gland infection, as previously described [27, 32]. Briefly, *Culicoides* were placed onto a glass microscope slide (Academy Science, UK), and heads were removed using a sterile 25G hypodermic needle (Fisher Scientific, UK) (one per individual) under a stereomicroscope. The heads and bodies of each individual were placed separately into collection tubes and processed as described above for homogenisation and detection of BTV RNA.

Due to low survival of *C. sonorensis* fed on calves at 21–35 dpi, additional naive *C. sonorensis* were provided with EDTA blood collected from the BTV‐3‐infected calves at either 21 dpi (calves 1, 3 and 4) or 22 dpi (calf 2) using a Hemotek membrane feeding system (Hemotek Ltd., UK) as previously described [26]. Moreover, additional collections of UK field‐caught *Culicoides* were offered a BTV‐3‐spiked blood meal via a Hemotek membrane feeding system or cotton pledgets [33] at a final viral titre of 10^6^ TCID_50_/mL. Blood meals were provided to *Culicoides* for 1.5 h before engorged female *Culicoides* were selected under light CO_2_ for incubation (as above). Surviving field‐caught *Culicoides* were dissected and processed individually for the presence of viral RNA in heads and bodies, as described above, while *C. sonorensis* was processed as whole bodies.

### 2.6. RNA Extraction and Real‐Time RT‐PCR

Total nucleic acid was extracted from 100 µL EDTA blood, tissue homogenate or *Culicoides* homogenate using the MagMAX CORE Nucleic Acid Purification Kit and the KingFisher Flex automated extraction robot according to the manufacturer’s instructions. BTV segment 10 RNA was amplified using an EXPRESS One‐Step Superscript quantitative real‐time RT‐PCR kit (Thermo Fisher Scientific, UK) as previously described [34], with modifications [15]. Briefly, 5 µL RNA was denatured at 95°C for 5 min, after which 15 µL reaction mix (consisting of 1x Express qPCR SuperMix Universal, 400 nM forward and reverse primers, 200 nM probe, 500 nM ROX passive reference dye, and 2 µL EXPRESS SuperScript Mix) was added. Cycling conditions consisted of 50°C for 15 min, 95°C for 20 s, followed by 45 cycles of 95°C for 3 s, 56°C for 30 s, and 72°C for 30 s. A 10‐fold serial dilution of BTV‐1 segment 10 RNA transcript was run alongside RNA extracted from each EDTA blood sample to quantify BTV genome copy numbers per millilitre of blood [35]. *Culicoides* with a *C*_*q*_ value of less than 25 and/or where BTV RNA was detected in dissected heads were considered to have developed a transmissible infection [27, 32], while *Culicoides* with any *C*_*q*_ following 8 days incubation were considered BTV RNA positive.

### 2.7. Virus Isolation and Titration From EDTA Blood Samples

EDTA blood collected from each calf at each dpi was immediately washed three times with PBS (by centrifugation; 1000 *g*, 5 min, room temperature) to remove any neutralising antibodies present and then stored at 4°C. Immediately prior to use in virus isolation or titration experiments, EDTA bloods were sonicated, as described previously [15], to release red blood cell‐associated BTV.

Virus isolation, onto KC cells, was attempted from washed, sonicated EDTA blood of each calf at all time points of the study where BTV RNA was detected in the blood, as previously described [15, 36]. A reduction in the *C*_*q*_ value of 3 or more between the inoculum and harvested material (collected 7 days following infection) was considered to indicate viral replication and therefore represent isolation of infectious BTV. To explore the limits of virus isolation as an indicator of potential transmissibility, a maximum of two further passages were attempted from the harvest material. If unsuccessful, virus isolation was attempted from the original EDTA blood sample up to a further two times, after which a negative isolation result was recorded.

Infectious viral titres were quantified in the blood (defined as 50% tissue culture infectious dose per millilitre blood [TCID_50_/mL]) of each calf at 7 dpi (and also 8 dpi for calf 1) by performing a 10‐fold end‐point titration of the washed, sonicated EDTA bloods on KC cells, as previously described [24].

### 2.8. Antibody Detection

BTV VP7 antibodies were detected in the serum of each calf at each dpi in duplicate using the ID Screen BT competition ELISA (Innovative Diagnostics, France) as per the manufacturer’s instructions. In addition, subclass (IgM and IgG)‐specific BTV VP7 antibodies were detected in serum in duplicate by indirect ELISA, as previously described [24]. Protective, neutralising BTV VP2 antibody titres were determined by the serum neutralisation test (SNT) using Vero cells, as described previously [24].

### 2.9. Haematology Analysis

Complete blood cell counts were analysed for each calf at −1, 2, 3, 4, 5, 6, 7, 8, 9, 10, 12, 14, 16, 18, 20, 21, 22, 24, 26, 28, 30, 34, 38, 42, 46, 50, and 56 dpi using a ProCyte Dx Haematology Analyser (IDEXX Laboratories Ltd., UK) according to manufacturer’s instructions.

### 2.10. Statistics

The proportion of BTV‐positive *Culicoides* (where a positive *Culicoides* is defined as one with any *C*_*q*_ value when tested after 8 days incubation at 25°C) after feeding was analysed using binomial generalised linear mixed models with a logit link function. Two models were constructed. In the first, the explanatory variables were *Culicoides* species (field‐caught or *C. sonorensis*), a quadratic function of dpi of the calf when feeding took place and an interaction between these, together with the calf as a random effect. In the second, the explanatory variables were *Culicoides* species, log_10_ genome copies/mL of blood in the calf at the time of feeding and an interaction between these, together with the calf as a random effect. In both cases, model simplification proceeded by stepwise deletion of non‐significant (*p* > 0.05) terms as judged by a likelihood ratio test. Statistical analysis was implemented using the lme4 package [37] in R (Version 4.4.0) (R Core [38]). Survival of *Culicoides* at different dpi was analysed using chi‐squared tests.

### 2.11. Ethics Statement

This in vivo study was carried out in accordance with the UK Animal Scientific Procedures Act (ASPA) 1986, which transposes European Directive 2010/63/EU into UK law. The study was approved by the UK Home Office under Project Licence (PPL) PP5796660 with moderate severity limits and humane endpoints derived from previous studies of different BTV strains at The Pirbright Institute [24, 26, 39]. All procedures were reviewed and agreed locally by the Animal Welfare and Ethics Review Board at The Pirbright Institute (AR001420).

## 3. Results

### 3.1. Infection of Calves With a UK BTV‐3 Isolate Using *C. sonorensis* Midges and Dynamics of Viraemia During BTV‐3 Infection

An average of 10 BTV‐3‐inoculated *C. sonorensis* (range: 3–16) were found to be fully or partially engorged following blood feeding on calves. All engorged and partially engorged *Culicoides* were BTV RNA positive at the time of feeding (Table 1), with all *Culicoides* considered to have a transmissible infection (i.e., a *C*_*q*_ value of less than 25). Only a single engorged female *C. sonorensis* with two partially engorged females was found to have blood‐fed on calf 1.

**Table 1:** Numbers of engorged and partially engorged BTV‐3 inoculated *C. sonorensis* which blood‐fed on each calf to establish infection.

Animal number	Number of fully engorged *Culicoides*	Number of partially engorged *Culicoides*	Mean *C*_*q*_ value of engorged *Culicoides* (range)
Calf 1	1	2	16.59 (16.23–16.97)
Calf 2	2	7	16.38 (15.85–16.60)
Calf 3	3	9	16.56 (15.81–17.04)
Calf 4	5	11	16.80 (16.05–17.49)

*Note:* Numbers of engorged and partially engorged *C. sonorensis* with detectable BTV RNA levels are given alongside the range of *C*_*q*_ values detected.

Detection of BTV RNA was possible in EDTA blood of all calves from 2 to 3 dpi (calves 2–4 and calf 1, respectively) with a similar pattern of viraemia observed between the individuals (Figure 1). All animals reached peak viraemia between 6 and 10 dpi (6 dpi for calf 4, 8 dpi for calf 2 and 10 dpi for calves 1 and 3), with similar viraemia levels reached in all calves, an average of 8.98 log_10_ BTV genome copies/mL blood (range: 8.76–9.28 log_10_ genome copies/mL blood; average *C*_*q*_ value: 20.15, range: 19.18–20.83). Despite only having one fully engorged *C. sonorensis* and fewest overall infected *Culicoides* acquiring a blood meal, calf 1 still developed viraemia (8.88 log_10_ genome copies/mL blood, *C*_*q*_ value: 20.49), reaching similar levels compared with the other calves, which received more infectious bites. Following peak viraemia, detectable levels of BTV RNA in the blood marginally declined to a plateau maintained up to 56 dpi, with an average of 7.26 log_10_ genome copies/mL blood remaining at this point (range: 7.00–7.86 log_10_ genome copies/mL blood; average *C*_*q*_ value: 25.59; range: 23.69–26.43).

**Figure 1:**
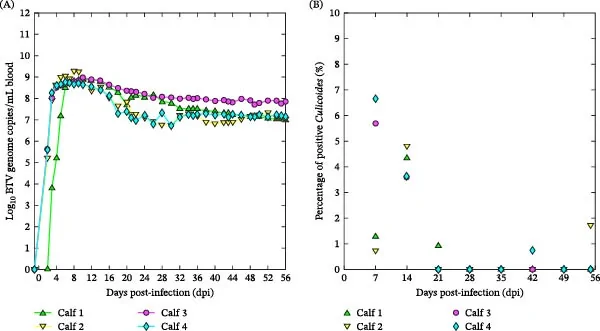
(A) Viraemia dynamics of four Holstein‐Friesian calves during infection with BTV‐3 UKG2023/04. Lines represent the log_10_ BTV genome copies per millilitre of blood. (B) Infection rates of *Culicoides* fed on sheep at times post‐infection. Symbols represent the proportion of positive *Culicoides* (UK field‐caught and colony *C. sonorensis* combined) after feeding on each infected calf and incubation for 8 days at 25°C.

### 3.2. UK BTV‐3 Strain Isolated From Calf Blood up to 56 dpi

Infectious BTV‐3 was isolated from the blood of calves 2, 3 and 4 from 2 dpi and from calf 1 from 5 dpi (Figure 2). Thereafter, infectious BTV‐3 was isolated at all time points up to 30 dpi across all four infected calves. However, beyond 24 dpi, often several attempts and/or passages of infected blood through KC cell monolayers were required to successfully isolate BTV‐3 from EDTA blood (Figure 2). Nevertheless, infectious BTV‐3 was isolated from EDTA blood of calves 2, 3 and 4 up to 56 dpi and from calf 1 up to 54 dpi (Figure 2).

**Figure 2:**
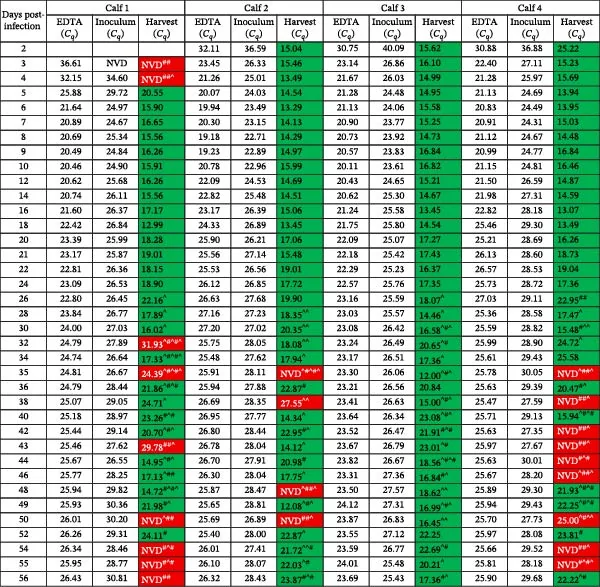
Isolation of BTV‐3 from EDTA blood of infected calves. Virus isolation was attempted from EDTA bloods of BTV‐3‐infected calves throughout infection to determine the duration BTV‐3 remains infectious in the blood. BTV real time RT‐PCR *C*_*q*_ values of EDTA calf blood, starting inoculum used to infect *Culicoides*‐derived KC cells and the harvest material (collected 7 days post‐infection) are shown. Successful (green) and unsuccessful (red). Samples requiring re‐passage = ^. Samples requiring re‐attempt from original blood = #. NVD, no virus detected.

Titration of EDTA blood collected from calves around peak viremia revealed that at 7 dpi, calf 2 had the highest infectious BTV titre (1.12 × 10^4^ TCID_50_/mL blood), followed by calf 4 with 6.31 × 10^3^ TCID_50_/mL blood and then calves 1 and 3 with the lowest titre of 3.55 × 10^3^ TCID_50_/mL blood. At 8 dpi, the infectious viral titre of calf 1 remained consistent with that of 7 dpi at 2 × 10^3^ TCID_50_/mL blood.

### 3.3. Clinical Signs Associated With BTV‐3 Infection in Holstein‐Friesian Calves

Calves 1 and 2 both presented with coughs and nasal discharge prior to infection, which persisted throughout most of the study. Following infection, all four Holstein‐Friesian calves displayed mild clinical signs typically associated with BTV infection, including coughing, nasal discharge, conjunctivitis and ulcers on the dental pad, lips and gums. The total clinical score showed no consistent increase over time for each animal (Figure S1). Mild oral ulceration/erosion was first observed in all calves from 7 to 15 dpi (Figure 3A). Lesions did not elicit excessive salivation or a loss of appetite. Conjunctivitis, including excess fluid around the eyes, was noted in three calves (Figure 3B) which progressed to an eye infection with ulceration and reddening of the cornea (Figure 3C) for calves 1 and 3 at 34 dpi, despite treatment with antibiotics. Calf 1 showed eventual improvement following treatment with 16.67% w/v cloxacillin benzathine (Orbenin, Zoetis). Calf 3 was also administered Orbenin, along with a 5‐day course of penicillin/streptomycin administered intramuscularly starting at 35 dpi and meloxicam (Metacam, Boehringer Ingelheim) (20 mg/mL) subcutaneously at 36 and 38 dpi. Further treatments of Orbenin were administered every other day between 50 and 54 dpi as well as daily bromfenac (Yellox, Croma) eye drops from 50 to 55 dpi.

**Figure 3:**
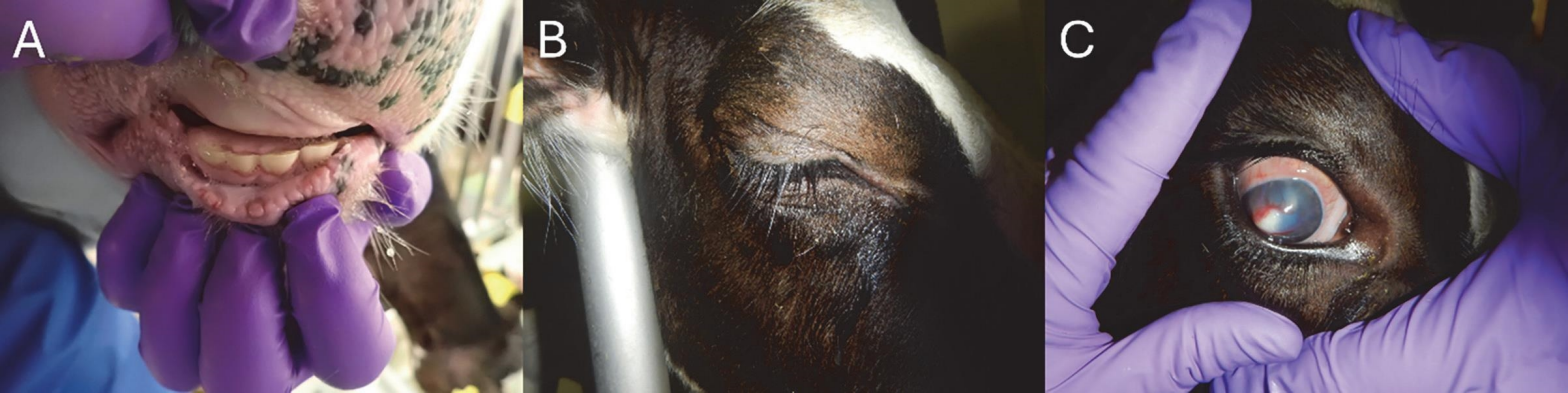
Clinical signs associated with BTV‐3 infection in British Holstein‐Friesian calves. Calves developed oral lesions on the dental pad, lips and gums (A) and signs of conjunctivitis with weeping eyes (B), which in one calf developed into an ulcerative eye infection (C).

Rectal temperatures of all four calves remained mostly consistent throughout the infection (Figure S2). Calf 3 had a raised temperature (1.05°C above baseline) at 4 dpi, which persisted for 1 day before returning to normal. Calf 2 experienced a raised temperature (1.35°C above baseline) approaching fever at 33 dpi, which again persisted for 1 day before returning to normal.

### 3.4. Onwards Transmission to *C. sonorensis* and UK Field‐Caught *Culicoides* spp.

A total of 7714 *Culicoides* (2295 UK field‐caught and 5419 *C. sonorensis*) acquired a blood meal from infected calves throughout the duration of the study. Of these, 59 *C. sonorensis* and 32 UK field‐caught *Culicoides* were found to have detectable levels of BTV RNA following incubation. Of the 59 *C. sonorensis* midges positive for BTV RNA, three were considered to have developed a transmissible infection (average *C*_*q*_ value: 16.31, range: 15.63–17.53), all of which were identified following feeding on calves around peak viraemia (7 dpi). Of the UK field‐caught *Culicoides* positive for BTV RNA, 20 were morphologically identified as the *C. obsoletus* group, seven as *Culicoides pulicaris* and five as the *Culicoides achrayi* group. One *C. obsoletus* group individual which fed on calf 4 at 6 dpi was considered to have a transmissible infection, with a whole body *C*_*q*_ value of 24.98 and viral RNA also detected in the head (*C*_*q*_ value of 29.35).

Detection of BTV RNA was consistently possible in incubated *Culicoides* acquiring a blood meal from the infected calves up to and including 21 dpi and 14 dpi for UK field‐caught and *C. sonorensis*, respectively. Thereafter, BTV RNA was detected in one *C. sonorensis* individual fed at 42 dpi (calf 4) and in two individuals fed at 55 dpi (calf 2), although these were not considered transmissible infections (*C*_*q*_ values of 38.14, 36.45 and 38.81, respectively).

The proportion of positive *Culicoides* depended on the dpi of the calf on which a midge fed, but the relationship differed significantly between UK field‐caught *C. sonorensis* midges (*p* < 0.001; Figure 4A). For field‐caught *Culicoides*, the proportion increased to a peak at around 12 dpi, after which it declined. By contrast, for *C. sonorensis*, the proportion declined from 7 dpi (i.e., the first time point at which feeding took place) but increased slightly at very late time points (after around 46 dpi) (Figure 4). This increase at later time points was attributed to the positive *Culicoides* fed at 42 and 55 dpi.

**Figure 4:**
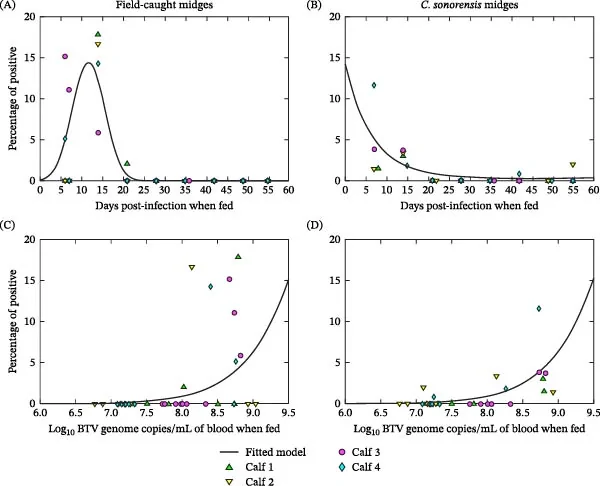
Proportion (%) of positive *Culicoides* after feeding on a BTV‐3‐infected calves. (A, B) The proportion of positive *Culicoides*, as dependent on days post calf infection on which they fed, for field‐caught (A) and *C. sonorensis* (B). (C, D) The dependence of proportion of positive *Culicoides* on log_10_ BTV genome copies/mL blood in the calf on which they fed, for field‐caught (C) and *C. sonorensis* (D). Symbols indicate the calf on which a *Culicoides* fed while the lines show the expected proportion positive based on the fitted binomial generalised linear mixed model.

The relationship between BTV genome copy number (log_10_ copies/mL blood) in a calf on which a *Culicoides* fed and the probability of it being positive did not differ significantly (*p* = 0.09) between *Culicoides* species. This dose–response relationship showed a significant (*p* < 0.001) increase in the probability of a midge being positive with increasing log_10_ BTV genome copies/mL of blood (Figure 4B), with a one log_10_ increase in BTV genome copies/mL of blood resulting in a 7.03‐fold (95% confidence interval: 4.31–12.68) increase in the odds of being positive.

A further 526 fully engorged *C. sonorensis*, which had fed on infected calves (~16 from each calf at each time point), were assessed for BTV uptake from infected animals immediately after feeding. Levels of BTV RNA detected in *C. sonorensis* processed immediately after blood feeding on infected calves were shown to decrease as calf infection progressed. *Culicoides* feeding on infected calves at 55 dpi acquired on average 4.9 *C*_*q*_ less (~1.5 log_10_ BTV genome copies/mL) than those feeding at peak viraemia (Figure S3A).

*C. sonorensis* were noted to have a significantly reduced survival rate following feeding on infected calves at days 21–35 post‐infection (*χ* ^2^ = 2233, *p* < 0.001; 15.37% (*n* = 2156) compared with 73.87% (*n* = 3263) survival; Figure S3B), meaning low numbers of insects were available for analysis for these time points. Therefore, additional *C. sonorensis* was provided with EDTA blood from all four calves from 21 to 22 dpi via an artificial membrane feeding system (Hemotek system, UK). A total of 1278 additional *C. sonorensis* successfully acquired a blood meal, of which 6 (1.21%) had detectable levels of BTV RNA following completion of the extrinsic incubation period. Survival of *C. sonorensis* fed with calf blood from 21 and 22 dpi via a Hemotek feeding system, was higher (38.4%) than those fed on infected animals at the same time point (14.43%), although still significantly lower than other time points (*χ* ^2^ = 490, *p* < 0.001, average: 73.87%, range: 59.13%–96.67%).

A further 536 field‐caught *Culicoides* acquired a BTV‐3‐spiked blood meal, with 321 individuals surviving incubation. A total of 40 *Culicoides* were positive for BTV RNA, an infection rate of 12.5%. Infected *Culicoides* were predominantly identified as the *C. obsoletus* group (*n* = 30; Table S1). The number of UK field‐caught *C. obsoletus* group individuals found to have a presumed transmissible infection (*C*_*q*_ < 25 and/or BTV RNA in the head) was low (0.68%). Field‐caught *Culicoides* tested immediately following blood feeding had an average *C*_*q*_ value of 31.52 (range: 29.94–35.01) in 24 fully engorged individuals.

### 3.5. Gross Pathology of BTV‐3 UKG2023 Infection in Holstein‐Friesian Calves

Pulmonary congestion (Figure 5A) was the most noted pathological abnormality observed in three of the four calves (calves 2–4). This was most severe in calf 3, accompanied by an indication of pulmonary oedema and frothing on the cut surface of the lung (Figure 5B) and in the trachea (Figure 5C). Calf 2 was the only individual to display classical pathological signs of BT with diffuse petechial haemorrhaging identified in the tongue, larynx, epiglottis and nasal mucosa proximal to the nares (Figure 5D–I). Small amounts of haemorrhaging in the parotid lymph node were noted in calf 4 (Figure 5I).

**Figure 5:**
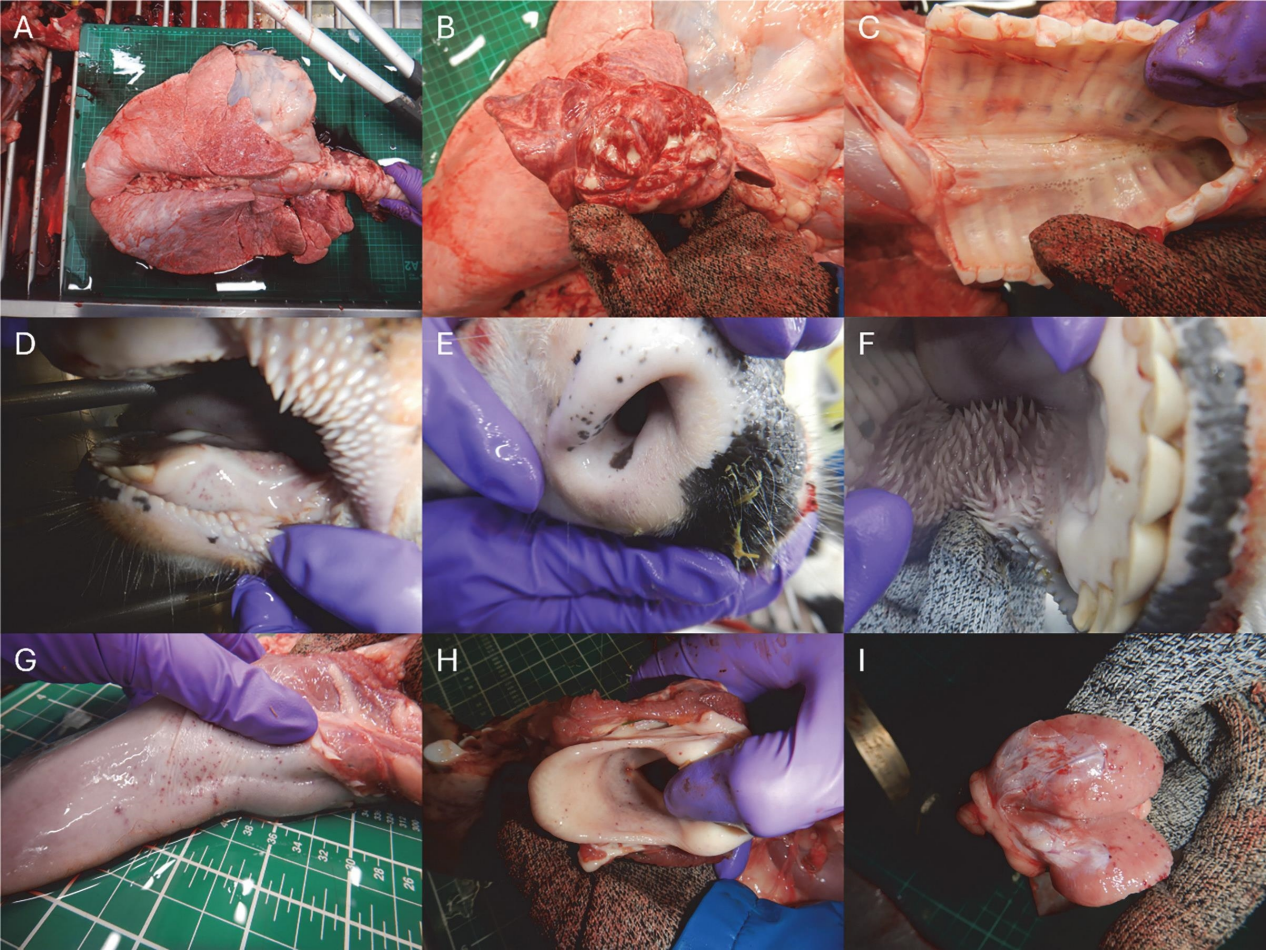
Gross pathological signs observed during BTV‐3 infection of four British Holstein‐Friesian calves at 56 days post‐infection. Congestion of the lung (A), accompanied by pulmonary oedema (B) and frothing in the trachea (C). Diffuse petechial haemorrhaging was noted on the lower gum (D) nares (E), pharynx (F), tongue (G), epiglottis (H) and in the parotid lymph node (I).

Despite no gross pathological abnormalities noted in the heart and spleen of any of the calves, BTV RNA was still detectable in both organs at 56 dpi (average *C*_*q*_ value: 34.13 and 33.21 for heart and spleen, respectively), while BTV RNA was also detectable in the parotid lymph node of calves 3 and 4 (Table S2). BTV RNA was not detected in any other organs that were tested.

### 3.6. BTV Antibody Dynamics

BTV VP7 antibodies were detected in the serum of all four BTV‐3‐infected calves, on average, from 10 dpi (Figure 6A). Calf 3 seroconverted earliest at 9 dpi, followed by calves 2 and 4 at 10 dpi and calf 1 at 12 dpi (Figure 6A). IgM VP7 antibodies were detectable first, followed by IgG VP7 antibodies (Figure 6B). IgM VP7 antibodies were detectable from 6 dpi in calf 2 and 3, from 7 dpi in calf 4 and from 9 dpi in calf 1. IgM VP7 antibodies peaked at 12 dpi for calf 2, 3 and 4, and 14 dpi for calf 1 and then gradually declined to a plateau at around 32 dpi (calves 2 and 4), 40 dpi (calf 1) and 34 dpi (calf 3), respectively (Figure 6B). Whilst detectable levels of IgM‐specific VP7 antibodies varied substantially across the four BTV‐3‐infected calves (Figure 6B), the dynamics of IgG VP7 antibodies were consistent. IgG VP7 antibodies were detectable from 18 dpi on average, with calves 3 and 4 class switching at 16 dpi, calf 2 at 18 dpi and calf 1 at 20 dpi. IgG VP7 antibodies increased steadily in all four calves to 56 dpi, reaching peak S/N ratios at 54 dpi (calf 4) or 55 dpi (calves 1, 2 and 3) averaging 46.70 (range 43.16 – 52.56). Protective, neutralising BTV VP2 antibodies were detectable in the BTV‐3‐infected calves, on average, from 14 dpi, with detectable VP2 antibodies from 10 dpi in calf 1, 14 dpi in calf 3 and from 16 dpi in calves 2 and 4 (Figure 6C). Neutralising antibody titres peaked at above 1/1280 in calf 4 at 35 dpi, much earlier than in calf 2 (54 dpi) and calf 3 (50 dpi). Calf 1 neutralising antibody titres peaked at 1/1280 at 50 dpi (Figure 6C).

**Figure 6:**
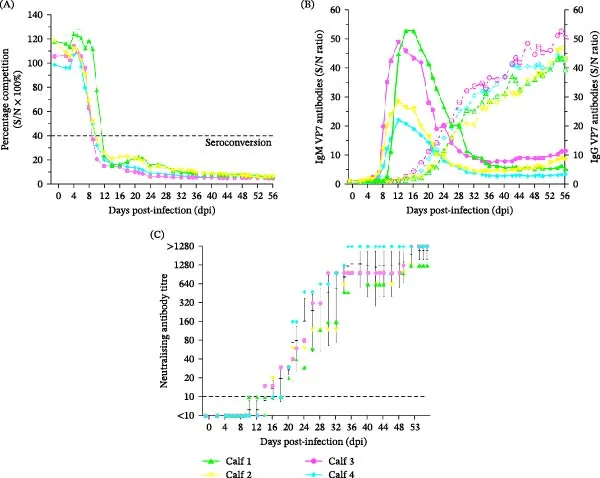
BTV antibody dynamics during BTV‐3 UKG2023/04 infection in Holstein‐Friesian calves. Seroconversion of BTV VP7 antibodies (A) and distinction of IgM (solid symbols and lines) and IgG (open symbols, hashed lines) BTV VP7 antibody dynamics (B). Neutralising BTV VP2 antibody dynamics (C).

### 3.7. Dynamics of Leukocytes During BTV‐3 Infection

Total leukocyte numbers declined around the peak of viraemia in all four BTV‐3‐infected calves, from ~2–4 dpi up to 7–14 dpi (Figure S4). This prominent lymphopenia appeared to specifically result from a consistent and prolonged decline in neutrophils in the blood from 2 to 5 dpi up to 14–28 dpi (Figure S4) and an acute decline in lymphocytes around the peak of viraemia. There was a very prominent post‐viraemia increase in lymphocytes, particularly for calves 1 and 3 (Figure S4). Eosinophils remained at low levels throughout the course of infection (Figure S4). Interestingly, monocyte numbers declined much earlier than other leukocytes during BTV‐3 infection (between ~3 and6 dpi), but this was later followed by a substantial increase, peaking between 14 and 20 dpi and thereafter declining to the end of the study (Figure S4).

## 4. Discussion

The emergence of BTV‐3 in northern Europe and its subsequent detection in the UK in November 2023 necessitated the characterisation of the BTV‐3 infection in British cattle. This study describes the infection of four British Holstein‐Friesian calves with this BTV‐3 strain through infectious bites received from *C. sonorensis*, the resultant viraemia, infection kinetics, clinical disease and onwards transmission to *Culicoides* vectors. Whilst calves developed mild clinical signs of BT, disease severity was lower than previously reported in UK sheep [15] and European cattle [8] infected with the same BTV‐3 strain. Infectious BTV‐3 was isolated consistently from all calves to 28 dpi and up to 56 dpi, with BTV RNA detected in *C. sonorensis* fed on these viraemic calves at 42 and 55 dpi. Together, this highlights the potential for cattle to act as a sub‐clinically affected reservoir of BTV infection for onwards transmission to competent *Culicoides* vectors for several weeks post‐infection.

In the current study, all four calves were infected with the BTV‐3 UKG2023/04 isolate, with BTV RNA detectable in the blood from 2 to 3 dpi until the end of the study at 56 dpi. Antibodies against the immunodominant BTV VP7 protein were consistently detected from 1 to 4 days after peak viraemia, whilst protective neutralising antibodies against BTV VP2 were detected in all calves on average from 14 dpi, similar to previous findings [2, 15]. The association of BTV particles with erythrocytes is hypothesised to facilitate their protection from the host immune response and result in the prolonged viraemia observed in animals [40]. Whilst detection of BTV RNA in ruminant blood has been demonstrated for up to 6 months post‐infection [17, 41], studies have indicated that persistence and detection of BTV RNA can extend beyond the length of infectious viraemia as determined by isolation on the cell culture [16, 17]. Therefore, understanding how BTV RNA detection in cattle blood correlates with the infection rate of *Culicoides* midges is essential to understand the epidemiological importance of cattle that remain positive by PCR for extended periods.

Some *C. sonorensis* individuals were shown to ingest sufficient virus from infected calves to initiate infection until the end of the study at 55 dpi, yet virus uptake within the blood meal was shown to decline as infection in cattle progressed. A small proportion of *Culicoides* vectors developed levels of infection considered sufficient for onwards transmission, all of which were identified following feeding on infected calves around peak viremia (6–7 dpi). Interestingly, BTV‐3 titres in the calves at 7 dpi were ~10^2^ TCID_50_/mL lower than those observed at the same time point in sheep infected with the same BTV‐3 strain ([15]; unpublished data). Despite these lower titres, BTV transmission from calves to *Culicoides* vectors was observed, in contrast to the absence of BTV transmission to *Culicoides* from BTV‐3‐infected sheep in a previous study [15]. Individual *C. sonorensis* blood‐fed on infected calves at 42 and 55 dpi were found to be infected with BTV‐3 following incubation, despite the presence of neutralising antibodies, and at time points where virus isolation from calf blood was possible but inconsistent in some animals. The low survival of *C. sonorensis* during incubation, which had fed on BTV‐3‐infected calves from 21 to 35 dpi, may have led to an underestimation of *Culicoides* infection rate, indicated by the increase in BTV detection following artificial feeding of *Culicoides* on the infected calf blood. Isolation of infectious virus from *C. sonorensis* fed on BTV‐17 infected cattle until 21 dpi has previously been reported, whilst also demonstrating the ability for *C. sonorensis* to acquire infection from ruminant blood with detectable levels of BTV RNA, yet below limits of virus isolation [17]. However, the low BTV RNA levels recovered from the BTV‐3‐positive *Culicoides* individuals are indicative of the acquisition of infectious virus, leading to sub‐transmissible infections. The virus acquisition threshold for the development of a fully disseminated, transmissible infection in *Culicoides* is likely higher than for midgut infection [42]. There remains, however, the possibility of BTV dissemination in an individual resulting from the acquisition of a few infectious virus particles. Our analyses indicate that a 10‐fold increase in BTV genome copies/mL blood would lead to a seven‐fold increase in the odds of infection in *Culicoides*.

Our laboratory estimates of the vector competence of UK *C. obsoletus* group midges for this BTV‐3 strain were low. This finding is contrary to an assessment of the high competence of the *C. obsoletus* and *C. pulicaris* group from the large proportion of positive pools (40%) collected at BTV‐3 outbreak sites in the Netherlands [43]. However, where the number of individuals in a pool was considered, a mean minimum infection rate of 1.4% was calculated. This, coupled with high *C*_*q*_ values (30–36) of most positive pools, is indicative of individuals with infections that may not have disseminated, potentially inflating the estimates of competence [43]. Infection rates in the present study are more in line with lower previous estimates for field prevalence in *Culicoides* during the 2006–2010 BTV‐8 outbreak in Europe [44–47] and are sufficient for the transmission of BTV‐3 in the UK. Nevertheless, the results presented here demonstrate that although the probability of transmission to vectors declines after peak viraemia, there remains a risk of onwards transmission to *Culicoides* vectors that may persist late into infection, up to at least 2 months. This would be especially pertinent under field conditions, where low infection rates are compensated for by the huge abundance and high biting rates of *Culicoides*. Further work is required to quantify the risk posed by insects feeding on infected cattle beyond peak viraemia and the contribution of extended host‐infected viraemia to the overwintering of BTV.

Isolation of infectious virus several weeks post‐infection has been demonstrated in cattle for other BTV serotypes/strains, including BTV‐8 and BTV‐17, until at least 39 and 49 dpi, respectively [17, 48]. However, the probability of detectable viraemia extending beyond 63 days is predicted to be low [16]. In the present study, virus isolation from calf blood was readily achieved up to 24 dpi, in agreement with previous studies [16, 17, 48]. After this time point, isolation of infectious BTV became more inconsistent yet was achieved on the first attempt at 34 and 52 dpi for two calves. Upon repeated passage, BTV was isolated until the end of the study at 56 dpi in three of the calves, demonstrating the presence of a virus able to infect a *Culicoides*‐derived cell line. This finding correlates with the observed transmission to *Culicoides* at up to 55 dpi. The relationship between erythrocyte association of BTV, host antibodies and the infectivity of BTV particles to *Culicoides* requires further investigation.

Importantly, calves became infected with BTV‐3 despite exposure to very low numbers of infectious bites (one fully and two partially engorged *C. sonorensis* for one calf), as demonstrated previously for sheep [26]. The number of infectious bites, or ‘dose’ has previously been suggested to determine the timing of the onset and peak of viraemia [48]. A previous in vivo study reported that one infectious bite may be sufficient to initiate infection, with a BTV strain‐dependent interaction between the number of *Culicoides* bites received and the onset of viraemia and maximum viral load [26]. In the present study, the magnitude and timing of peak viraemia in infected calves were independent of the number of infectious bites received. Whilst viraemia was similar between calves, detection of BTV RNA in the blood in the calf receiving the fewest infectious bites was 1 day later than in the other calves. Whilst it appears a single infectious *Culicoides* bite may be sufficient to initiate infection causing disease, further work is required to understand the complex interplay between the BTV strain, the number of infectious bites or virus doses received and the resulting infection dynamics.

Cattle are considered a reservoir host of BTV, as infection is common and typically sub‐clinical [49]. The severity of clinical disease associated with BTV infection in cattle can vary depending on the inoculation route, virus serotype/strain and the breed and age of the host animal [3]. Following the emergence of BTV‐3 in the Netherlands, severe clinical disease was reported in cattle, accompanied by decreased milk yield and increased mortality at a national level [50]. Indeed, a study of five farms during the BTV‐3 outbreaks in the Netherlands reported that the percentage of cattle showing clinical signs varied from 8.1% to 50.4% per farm [50]. The severity of disease in cattle in the field varied from sub‐clinical and mild disease to severe cases, particularly in calving cows [50]. Further investigations into the effects of the BTV‐3 outbreak on cattle health in the Netherlands found a significant rise in abortions, premature births and calf mortality associated with BTV‐3 infection [8]. The impact of BTV‐3 infection on cattle reproduction in the UK has yet to be quantified, although cases of abortions, premature births and calf mortalities were reported in early 2025 following BTV infections in 2024.

All four calves in the current study presented with mild clinical BT disease following infection, including conjunctivitis, nasal discharge and mild transient elevation in temperatures, similar to previous studies with BTV‐8 infected calves [2]. All BTV‐3‐infected calves developed mild mouth ulcers/erosions, visible under close observation. Importantly, these signs did not impact the appetite of the calves and did not result in excessive salivation or drooling. Two calves developed conjunctivitis, which in one calf progressed into an ulcerative eye infection despite antibiotic treatment. Due to the late presentation in this study, it is unclear whether BTV‐3 was the aetiological agent for the corneal ulceration or if it resulted from a secondary infection complication or co‐morbidity worsened by BTV‐3 infection. Nevertheless, the clinical signs noted here would likely go undetected on large farms unless animals were under close and constant observation. The individual variation in signs and severity of BT disease [3, 50] suggest that greater infection rates in the field, as seen in the Netherlands, may increase the probability of individuals exhibiting notable clinical disease. Further investigation is required to identify factors that may determine differences in the observation and severity of clinical disease between regions affected by BTV‐3.

Time to seroconversion of BTV VP7 antibodies in cattle was consistent with that previously reported for BTV‐3‐infected sheep [15], in line with their comparable onset and dynamics of viremia. Whilst there are few published studies investigating antibody dynamics in BTV‐infected cattle in vivo, Darpel et al. [2] demonstrated comparable time to seroconversion (8–12 dpi) in cattle needle‐inoculated with a European BTV‐8 strain. Sheep infected with BTV‐8 FRA2017 also demonstrated a similar time to seroconversion [39], notably later than a more pathogenic UK BTV‐8 strain. Whilst IgM‐specific VP7 antibody responses were fairly comparable between BTV‐3‐infected cattle and sheep, the IgG‐specific VP7 antibody responses were of much higher S/N ratios by 28 dpi in cattle than in sheep [15] and in sheep, these IgG VP7 S/N ratios have been shown to approximately correlate to antibody quantity [24]. It has yet to be investigated how comparable the avidity of these IgG VP7 antibodies is between host species infected with this UK BTV‐3 strain. A previous study identified a delay in the production of affinity matured IgG‐specific anti‐VP7 antibodies and virus neutralising (anti‐VP2) antibodies in BTV‐8 NET2006‐infected sheep compared to sheep infected with an attenuated BTV‐8 strain, resulting from the destruction of follicular dendritic cells in germinal centres affecting B cell division [51]. It would be important to explore whether BTV‐3 also disrupts germinal centres in infected sheep and cattle to understand whether such mechanisms are responsible for these observed differences in antibody dynamics between host species.

Protective neutralising (anti‐VP2) antibody dynamics and titres in the BTV‐3‐infected calves were comparable to those observed in clinically affected adult sheep infected with the same BTV‐3 strain [15]. Whilst neutralising antibodies against BTV VP2 are a known correlate of protection in ruminants (from reinfection with strains of a homologous serotype), the role of cellular immunity during BTV infection has been understudied, particularly in the context of comparative immunology between susceptible host species. Several studies have shown a potential role for T‐cell‐mediated immunity in protection from BTV [24, 52–54]. Further work is required to understand whether cellular immune responses differ between infected cattle and sheep and whether this could explain the differences in their clinical disease presentation. Our work here demonstrates that cattle develop the characteristic lymphopenia widely observed at peak viremia during BTV infection in ruminants [15, 55, 56], with an increase in lymphocyte numbers detected ~2–3 weeks post‐viremia most likely corresponding to an increase in CD8^+^ T cells [24, 57, 58]. The pronounced and uniform increase in monocytes observed in the blood around peak viremia in BTV‐3‐infected cattle was not previously observed in sheep infected with the same strain, except for the one clinically mild sheep which demonstrated an increased monocyte count from 7 dpi [15]. A previous in vivo study also showed no significant changes in blood monocyte counts in BTV‐1‐ and BTV‐8 needle‐inoculated sheep up to 15 dpi [55]. Together, this may suggest a potential correlation of monocyte‐induced immunity with more favourable clinical outcomes; however, further work is required to investigate their role during BTV infection. A consistently low eosinophil count was observed in the calves, a marked difference to the substantial changes previously reported in BTV‐3‐infected sheep [15]. Further work is required to understand the implications of this finding and/or the underlying causes.

## 5. Conclusions

*Culicoides*‐initiated BTV‐3 infection in four Holstein‐Friesian calves elicited mild clinical BT disease. The number of infectious bites calves received did not influence clinical signs observed or the magnitude or timing of peak viraemia. The BTV genome was detected in *C. sonorensis* individuals fed on infected calves at up to 55 dpi, and the virus was isolated from infected calves until the end of the study, at 56 dpi, although this became more challenging beyond 24 dpi. Whilst the probability of onwards transmission to *Culicoides* vectors declines after peak viraemia, a non‐negligible risk may remain for a substantial time post‐infection.

## Funding

This study is supported by the Department for Environment, Food and Rural Affairs, UK Government (Grants SE 4117 and SE 2623) and Biotechnology and Biological Sciences Research Council (Grants BBS/E/PI/230001B, BBS/E/PI/230002B, BBS/E/PI/23NB0003, BBS/E/PI/23NB0004 and BBS/E/PI230002C).

## Conflicts of Interest

The authors declare no conflicts of interest.

## Supporting Information

Additional supporting information can be found online in the Supporting Information section.

## Supporting information


**Supporting Information** Supporting data provided includes clinical scores, temperature and white blood cell counts of BTV‐3‐infected calves (Figures S1, S2 and S4). Quantification of BTV‐3 uptake and survival of *Culicoides* fed on the calves over time is presented (Figure S3). Figure S1: Clinical scores of four Holstein‐Friesian calves infected with BTV‐3 UKG2023/04. Figure S2: Rectal temperatures of four Holstein‐Friesian calves infected with BTV‐3 UKG2023/04. Figure S3: Uptake of BTV‐3 by *Culicoides* (A) and survival (B) following blood‐feeding on infected Holstein‐Friesian calves throughout the course of infection. *C*_*q*_ values of *C. sonorensis* processed immediately following blood‐feeding on BTV‐3 infected calves at each time point to demonstrate viral uptake within the bloodmea (A). Horizontal black lines indicate median, 25^th^ and 75^th^ percentiles, whiskers extend to the largest and smallest values within 1.5 times the interquartile range from the 25^th^ and 75^th^ percentiles, closed dots indicate outliers. Survival of UK field‐caught and colony‐reared *Culicoides* following feeding on infected calves at each time point post‐infection (B). Error bars indicate mean ± SD. Figure S4: White blood cell counts during BTV‐3 UKG2023/04 infection in Holstein‐Friesian calves. The number and species of field‐collected *Culicoides* found to be RT‐PCR positive for BTV‐3 following feeding on infected cattle (Table S1) and the detection by RT‐PCR of BTV‐3 RNA in tissues of calves collected at post‐mortem are provided (Table S2).

## Data Availability

The data that support the findings of this study are available from the corresponding author upon reasonable request.
