# Supplementary material for: Infection Dynamics and Clinical Signs of Bluetongue Virus Serotype 3 in Cattle and Transmission to Culicoides Vectors
Source: Transbound Emerg Dis. 2026 Oct 31;2026:9342380. doi: 10.1155/tbed/9342380 (PMC13723317; doi:10.1155/tbed/9342380)
Supplement: Supplementary file 1 [file TBED-2026-9342380-s001.zip › Table S1.docx]

**Table S1. Numbers of UK field-caught Culicoides with detectable BTV RNA levels following a BTV-3 spiked bloodmeal and incubation at 25^o^C for eight days.** Numbers of field-caught Culicoides with detectable BTV RNA levels and with a RT-PCR C_q_ value <25 (indicative of transmissible infection) are given alongside the range of C_q_ values detected.

| ***Culicoides* species** | **Number of *Culicoides* tested** | **Number of positive *Culicoides*** | **Number of positive *Culicoides* with C_q_ <25** | **Mean C_q_ values (range)** |
| --- | --- | --- | --- | --- |
| ***C. obsoletus* group** | 292 | 30 | 1 | 35.14 (22.37 – 40.09) |
| ***C. pulicaris*** | 14 | 8 | 0 | 36.85 (32.64 – 41.90) |
| ***C. achrayi* group** | 3 | 1 | 1 | 22.98 |
| ***C. festivipennis*** | 10 | 1 | 0 | 36.85 |
