# Supplementary material for: Infection Dynamics and Clinical Signs of Bluetongue Virus Serotype 3 in Cattle and Transmission to Culicoides Vectors
Source: Transbound Emerg Dis. 2026 Oct 31;2026:9342380. doi: 10.1155/tbed/9342380 (PMC13723317; doi:10.1155/tbed/9342380)
Supplement: Supplementary file 1 [file TBED-2026-9342380-s001.zip › Table S2.docx]

**Table S2. Detection of BTV RNA in tissue samples collected from four BTV-3 infected calves post-mortem at 56 dpi by real-time RT-PCR.** BTV RNA levels (C_q_ values) detected in heart, spleen and the parotid lymph node are given. NVD = no virus detected.

|  | **Heart** | **Spleen** | **Parotid lymph node** |
| --- | --- | --- | --- |
| **Calf 1** | 36.22 | 33.99 | NVD |
| **Calf 2** | 33.50 | 34.51 | NVD |
| **Calf 3** | 33.21 | 33.53 | 44.33 |
| **Calf 4** | 33.57 | 30.81 | 29.01 |
